# Expanding the classical paradigm: what we have learnt from vertebrates about sex chromosome evolution

**DOI:** 10.1098/rstb.2020.0097

**Published:** 2021-09-13

**Authors:** Lukáš Kratochvíl, Matthias Stöck, Michail Rovatsos, Mónica Bullejos, Amaury Herpin, Daniel L. Jeffries, Catherine L. Peichel, Nicolas Perrin, Nicole Valenzuela, Martina Johnson Pokorná

**Affiliations:** ^1^ Department of Ecology, Faculty of Science, Charles University, Viničná 7, Prague, Czech Republic; ^2^ Leibniz-Institute of Freshwater Ecology and Inland Fisheries - IGB (Forschungsverbund Berlin), Müggelseedamm 301, 12587 Berlin, Germany; ^3^ Amphibian Research Center, Hiroshima University, Higashi-Hiroshima 739-8526, Japan; ^4^ Department of Experimental Biology, Faculty of Experimental Sciences, University of Jaén, Las Lagunillas Campus S/N, 23071 Jaén, Spain; ^5^ INRAE, LPGP, 35000 Rennes, France; ^6^ State Key Laboratory of Developmental Biology of Freshwater Fish, College of Life Sciences, Hunan Normal University, Changsha 410081, Hunan, People's Republic of China; ^7^ Department of Ecology and Evolution, University of Lausanne, CH-1015 Lausanne, Switzerland; ^8^ Institute of Ecology and Evolution, University of Bern, CH-3012 Bern, Switzerland; ^9^ Department of Ecology, Evolution, and Organismal Biology, Iowa State University, Ames, IA 50011, USA; ^10^ Institute of Animal Physiology and Genetics, Czech Academy of Sciences, Rumburská 89, Liběchov, Czech Republic

**Keywords:** evolution, sex chromosomes, sex determination, vertebrates

## Abstract

Until recently, the field of sex chromosome evolution has been dominated by the canonical unidirectional scenario, first developed by Muller in 1918. This model postulates that sex chromosomes emerge from autosomes by acquiring a sex-determining locus. Recombination reduction then expands outwards from this locus, to maintain its linkage with sexually antagonistic/advantageous alleles, resulting in Y or W degeneration and potentially culminating in their disappearance. Based mostly on empirical vertebrate research, we challenge and expand each conceptual step of this canonical model and present observations by numerous experts in two parts of a theme issue of *Phil. Trans. R. Soc. B.* We suggest that greater theoretical and empirical insights into the events at the origins of sex-determining genes (rewiring of the gonadal differentiation networks), and a better understanding of the evolutionary forces responsible for recombination suppression are required. Among others, crucial questions are: Why do sex chromosome differentiation rates and the evolution of gene dose regulatory mechanisms between male versus female heterogametic systems not follow earlier theory? Why do several lineages not have sex chromosomes? And: What are the consequences of the presence of (differentiated) sex chromosomes for individual fitness, evolvability, hybridization and diversification? We conclude that the classical scenario appears too reductionistic. Instead of being unidirectional, we show that sex chromosome evolution is more complex than previously anticipated and principally forms networks, interconnected to potentially endless outcomes with restarts, deletions and additions of new genomic material.

This article is part of the theme issue ‘Challenging the paradigm in sex chromosome evolution: empirical and theoretical insights with a focus on vertebrates (Part II)’.

## 1. The ‘canonical’ scenario of sex chromosome evolution

**Figure 1.**
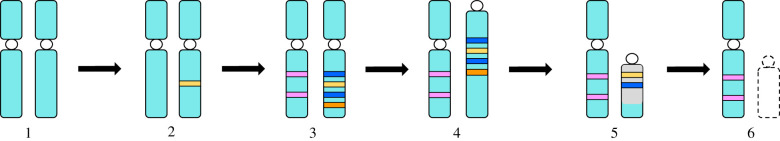
Canonical scenario of sex chromosome evolution. (1)—ancestral autosomes; (2)—emergence of a sex-determining gene (yellow); (3)—accumulation of sexually antagonistic/advantageous alleles (blue, pink) and/or deleterious recessive alleles (orange); (4)—cessation of recombination, depicted by a pericentromeric inversion changing the chromosome shape from metacentric to acrocentric as one potential example; (5)—degeneration of sex-specific (Y or W) chromosomes, i.e. accumulation of deleterious mutations, gene loss and accumulation of repeats and heterochromatin; (6)—loss of the entire sex-specific sex chromosome. For simplification, only the heterogametic sex is depicted.

Sex chromosomes evolved many times independently in eukaryotes and are one of the best examples of convergence at the genomic level. Until recently, it was generally assumed that sex chromosome evolution follows a canonical one-way trajectory (figure 1). This model, which aims to explain the evolution of differentiated sex chromosomes, has been formed over roughly 100 years since the seminal paper by Muller [1].^1^ According to this now widely accepted scenario, sex chromosomes evolved from a pair of autosomes, which acquire a sex-determining locus [2]. If this locus possesses a dominant sex-determining allele, or an allele whose function is dosage dependent, the genotype at this locus will now determine the sex of its bearers, and one allele will become restricted to a single sex. Furthermore, any loci that are genetically linked to the now sex-limited allele have a lower chance of being present in the opposite sex. Such a scenario is advantageous in the case of sexually antagonistic loci, which possess alleles that are beneficial (e.g. by shifting a trait expressed in both sexes towards the phenotypic optimum of the particular sex) or essential (e.g. controlling proteins exclusively used in sperm development) for one sex but detrimental for the other sex. Theoretically, selection should favour the suppression of recombination between the sex-determining locus and such sexually antagonistic loci within a sex chromosome to ensure the alleles of each occur in their appropriate combinations and resolve genomic conflict. Under the canonical model, following Fisher's early work published in 1931, this process is considered to play a major role in the progressive loss of recombination and subsequent specialization of sex chromosomes in the control of sex-specific phenotypes [3–5]. In the absence of recombination, the non-recombining regions of sex-specific chromosomes (Y or W) start to accumulate various repetitive elements and deleterious mutations owing to increased Hill–Robertson interactions and Muller's Ratchet [6,7]. This leads canonically to the progressive loss of genes or gene function (in turn resulting in unequal numbers of functional copies of many genes between the sexes) and potentially to structural changes such as deletions and heterochromatinization. The sex-specific sex chromosome can thus progressively degenerate and ultimately might even disappear from the genome entirely [8,9].

This canonical model is explicitly described or depicted in many presentations, textbooks and papers in the field, and strongly influences various contemporary ideas about sex chromosome evolution (e.g. [8–15]). However, although this model no doubt encompasses many important processes and drivers of sex chromosome evolution, several of its main components remain controversial, oversimplified or still lack empirical evidence. With this in mind, in this theme issue we have tried to stimulate discussion on each major component of the canonical model and chose contributors and framed topics to think beyond. Given the wealth of data that have been generated in the field over the past several years across a large number of vertebrate taxa, we felt that now is the perfect time for reevaluating old hypotheses and, if the data demand, replacing them with new ones. The studies comprise two parts of a theme issue of the *Phil. Trans. R. Soc. B.*, and were chosen to celebrate, challenge and expand the existing paradigm of sex chromosome evolution, largely from a vertebrate research perspective. This focus reflects our opinion that crucial questions can be best tackled in a well-studied monophyletic group with high variability in sex determination (reviewed in [16]) and good knowledge on gene functions in key developmental pathways in several model species. We hope this collection will inspire experts in other lineages and contribute to research progress on sex determination and sex chromosome evolution.

In this opening review, we highlight aspects of the canonical model where we believe fine tuning, modification, expansion or a complete reconsideration is warranted. Specifically we focus on the following aspects of the classical scenario (figure 1): (i) What was the situation prior to the origin of sex chromosomes? Why do some lineages apparently not have sex chromosomes? (ii) What is the first evolutionary step to make a sex chromosome, i.e. what are the origin and function of sex-determining loci? (iii) Is sexually antagonistic selection generally important for the evolution of sex chromosomes? (iv) Which mechanisms are responsible for the cessation of recombination in sex chromosomes? (v) Is the differentiation pathway indeed unidirectional, i.e. from poorly to highly differentiated/degenerated sex chromosomes or (vi) even loss of Y/W? Moreover, we briefly tackle the question of what are the general consequences of possessing (differentiated) sex chromosomes. We also explore the variation in the differentiation of sex chromosomes under male (XX/XY) and female (ZZ/ZW) heterogamety, and whether, ultimately, particular genomic parts (blocks, loci) are more frequently co-opted for a role in sex chromosomes.

## 2. What was the situation prior to the origins of sex chromosomes under the canonical model? Why are sex chromosomes absent in some lineages?

In gonochoristic organisms, sex determination (for terminology see the Glossary at [16]) is a crucial process, affecting individual and thereby population genetics, viability and evolution. Similarly, deciding when, how and under which conditions to switch sex in sequential hermaphrodites is also central. There is a surprising diversity and variability in sex determination mechanisms among vertebrates. Certain gonochoristic lineages rely on environmental sex determination (ESD), lacking consistent genotypic differences between males and females. On the other hand, in genotypic sex determination (GSD), males and females differ in parts of their genomes (sex chromosomes), from single nucleotide differences to large hemizygous chromosomal regions (or even germline specific chromosomes). Therefore, ESD may be viewed as a special case of polyphenism, i.e. the process where alternative morphs—here males and females—are set by particular environmental cues, triggering epigenetic mechanisms. GSD may be considered as genetic control of alternative discrete phenotypes [12,17]. The dichotomy between GSD and ESD remains controversial and several authors, including some of us, view pure ESD and pure GSD as the most extreme ends of a continuum of sex determination systems [18–21]. The debate concerns the question how to classify environmental influence on sex ratios and even gonadal development, caused for example by sex-specific mortality and fertilization, maternal effects (e.g. maternal hormones in the ovum) and environmentally induced sex reversals. Some authors interpret such mixed systems as an evidence for an ESD–GSD-continuum, while proponents of the dichotomy believe that it is useful to distinguish between true ESD and environmentally dependent sex ratio under GSD [12,22]. This idea reflects the view that ‘the continuous phenotypic pattern of sex-determining systems is generated by a discrete, dichotomous underlying process' [21, p. 680]. Regardless of these differences in classification, even sporadic environmentally induced sex reversals may have important consequences for the differentiation of sex chromosomes ([23–25]; see the part about the ‘fountain of youth’ model in §4).

**Figure 2.**
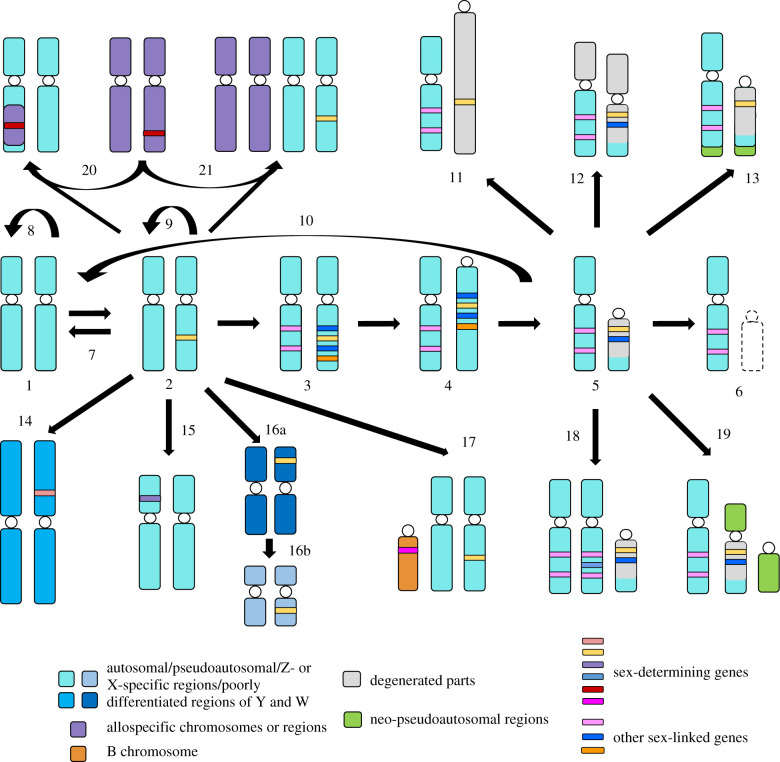
Overview of the steps in sex chromosome evolution with empirical support in vertebrates. This hypothetical network of evolutionary trajectories may branch off to potentially endless outcomes with a possibility to freeze for a long time and even reverse to certain states. Steps (1–6) are the same as in figure 1 but sexually antagonistic genes are not necessarily involved and can be replaced here by general sex-linked genes; (7)—switch to hermaphroditism or ESD, where no sex chromosomes are present; (8)—long-term evolution without emergence of sex chromosomes; (9)—long-term persistence of poorly differentiated sex chromosomes; (10)—reversal to stages with less differentiated sex chromosomes; (11)—expansion of repeats on sex-specific sex chromosome causing its change in size; (12)—accumulation of repeats on both sex chromosomes; (13)—fusion of the sex chromosomes with an autosome leading to expansion of the pseudoautosomal region; (14)—emergence of a new sex-determining locus on another chromosome; (15)—emergence of a new sex-determining locus within existing sex chromosomes; (16a,b)—two translocations of the same sex-determining locus to other chromosomes; (17)—origin of a new system of sex determination by involvement of B chromosome; (18)—emergence of sex-determining systems with three homologous sex chromosomes; (19)—fusion of sex chromosomes with an autosome leading to multiple neo-sex chromosomes; (20)—introgression of a sex-determining gene from a different population or species; (21)—allopolyploidization connected with emergence of a new sex-determining system in a genome of hybrid-origin. For simplification, only the heterogametic sex is depicted.

In any case, a common GSD mechanism is by sex chromosomes (polygenic sex determination in vertebrates or haplo-diploid sex determination of some mites, insects and rotifers can be considered GSD without sex chromosomes; their classification depends on a definition of sex chromosomes). The classical paradigm starts with a pair of autosomes, ready to become sex chromosomes in the next step (rarely, sex chromosomes might evolve from B chromosomes, i.e. genomic elements with a non-Mendelian inheritance present in different numbers among members of a population; e.g. [26,27]; case 17 in figure 2). But who was this enigmatic ancestor possessing such autosomes? Basically, it may already have been a GSD species and hence possessed sex chromosomes, but not necessarily. Sex chromosomes may also evolve *de novo* in an ancestor without sex chromosomes, mainly with hermaphroditism or ESD. In angiosperms, gonochorism evolved mostly from the ancestral simultaneous hermaphroditism [10], while in vertebrates, simultaneous hermaphroditism is extremely rare and very likely a derived condition. Likewise, sequential hermaphroditism, which occurs in teleost fishes, is also likely a derived condition [28,29] and tends to return to gonochorism [29]. Ohno [2] suspected that GSD (and thus sex chromosomes) evolved in amniotes multiple times independently from ancestral ESD. This hypothesis received support from research on squamate reptiles [12,22,30] and other sauropsids (i.e. the lineage including reptiles and birds), such as turtles [12,31,32], which points to non-homology of sex chromosomes across amniote GSD lineages (but see e.g. [33], critically discussed in [34]). Recently, Straková *et al*. [35] suggested that ESD in amniotes evolved from ancestral sequential hermaphroditism, which turned into ESD via a heterochronic shift, that is, by moving the timing of the ontogenetic period of sex change from the adult to the embryo. Subsequently, the loss of responsiveness to environmental stimuli led to GSD, where sex is typically decided already at conception [21], i.e. GSD comprised another heterochronic shift in the timing of the decision about individual sex [35]. This scenario is based on similarities of sequential hermaphroditism and ESD, such as the absence of sex differences in genomes, biased population sex ratios and potentially also molecular epigenetic mechanisms related to general stress responses [36–38]. If further supported, the evolution of GSD in some vertebrates and angiosperms may share unexpected similarities. Namely, sex chromosomes may then have evolved primarily to suppress the function of one sex and enhance that of the opposite sex, compared to the ancestral situation without sex chromosomes.

It will be fascinating to further uncover the molecular (epigenetic) mechanisms of sex change in vertebrate sequential hermaphrodites [39]. Despite recent progress, molecular mechanisms of sex determination in ESD species [36,40–42] remain underexplored and studied in only a few organisms, as are the molecular changes connected with transitions from hermaphroditism/ESD to GSD (and *vice versa*). Piferrer [37] explores the importance of epigenetics in transitions among sex determination systems and how epimutations could facilitate genetic changes accompanying and stabilizing a new sex determination mechanism. Up to now, we know too little about the epigenetic changes required in gonadal developmental pathways that allow such transitions.

There are several potential reasons why gonochorism or sequential hermaphroditism appear to be advantageous compared to simultaneous hermaphroditism. For example, they allow for selfing-avoidance and efficient specialization of an individual in a given time to a sex-specific function. Indeed, this might explain why simultaneous hermaphroditism is so rare among vertebrates [29]. However, it is less clear why some lineages rely on sex chromosomes while others do not. Sequential hermaphroditism and ESD can potentially enhance individual fitness under given environmental/social conditions [43,44]. In most cases, sex chromosomes ensure or even enforce stable Darwinian–Fisherian sex ratios, i.e. ratios leading to equal parental expenditure in offspring of both sexes [45,46], and may help solve the intralocus sexual conflict over the expression of a trait [3–5]. On the other hand, female-biased sex ratios in a population, as seen in many protogynous sequential hermaphrodites and ESD species [35], can be advantageous for population growth (although opposed by individual selection), reducing the two-fold costs of sex. This cost stems from the fact that males cannot themselves produce offspring, and thus a sexual population with a 50 : 50 sex ratio grows at half the rate of an (all-female) parthenogenetic population [47]. Populations with female-biased sex ratios possess lower costs of males for population growth. Beyond their roles in sex determination (see §3), sex ratios (see earlier in this section) and sexually antagonistic selection (see §4), sex chromosomes may profoundly affect individual fitness, population viability and long-term evolution. Differentiated W and Y sex chromosomes may lower fitness by increasing mortality, decreasing longevity and contributing to failures in gametogenesis or gamete loss [48–50]. Decreased longevity in the heterogametic sex in species with differentiated sex chromosomes was explained by several mechanisms including the ‘unguarded X/Z’, ‘toxic Y/W’ hypotheses and conflict between the paternally transmitted Y chromosome and mostly maternally transmitted mitochondria (mother's curse) [51]. The ‘unguarded X/Z’ hypothesis states that the heterogametic sex suffers higher mortality, as any effect of (mostly recessive) negative mutations at hemizygous X- and Z-linked loci is, in contrast to the homogametic sex, not masked by a second, functional copy [1,52]. The ‘toxicity’ hypothesis suggests that accumulation of Y- and W-specific active transposable elements could lead to sex-biased transposition and genome instability, likely detrimental to genome and organism [53,54], while in the long run potentially increasing evolutionary plasticity. Classically, transposable elements are assumed to have accumulated in the non-recombining regions of sex chromosomes after the cessation of recombination. However, they may also contribute to the rise of sex-determining genes during the birth of sex chromosomes and rewiring of gonadal differentiation networks [55]. Moreover, they might also be involved at the very beginning of the cessation of recombination of sex chromosomes, as their activity can dramatically change local recombination rates. The density of transposable elements is often negatively correlated with recombination, although this pattern is not universal [56]. Therefore, without further testing, it is difficult to disentangle what came first: the suppression of recombination, or the accumulation of transposable elements. The answer may not be the same in all cases. Nonetheless, transposable elements affect processes on sex chromosomes and in turn the entire genome. Sex linkage also affects rates of molecular evolution, sometimes so profoundly that their signatures may reflect past sex linkage in a region that has again become autosomal. Such instances may be applied to reconstruct the history of sex chromosome systems [57]. The effect of the rates of molecular evolution of sex-linked loci on clade evolvability deserves more attention.

Sex chromosomes can also profoundly affect lineage diversification rates ([58]; but see [59]). Haldane's rule [60], which states that the heterogametic sex of a hybrid is more often less fit (inviable, sterile, less fertile), has received much support across animals and plants. It is thus well established that differentiated sex chromosomes contribute significantly to speciation and affect hybridization and introgression [61–63]. However, these effects remain largely unexplored in systems with poorly differentiated sex chromosomes [64]. Haldane's effects may lead to less introgression of loci linked to well-differentiated sex chromosomes across hybrid zones in comparison to autosomes. By contrast, undifferentiated sex chromosomes might be more susceptible to introgression and may contribute to the emergence of multi-locus systems [65] or other derived sex determination systems [66] (case 20 in figure 2). Hybrids of parental species with higher divergence may exhibit sex-specific distortions of gametogenesis typified by potential male sterility and frequently female clonality, often closely linked to polyploidization [67–69]. Most of these hemiclonal, clonal and meroclonal (Glossary in [64]) vertebrates of hybrid-origin evolved in parental GSD lineages with undifferentiated sex chromosomes under male (XY) or—probably more frequently—female heterogamety (ZW) [64]. The emergence of a new sex-determining system might be linked to allopolyploidization [64,70] (case 21 in figure 2). Improving our understanding of the genomic preconditions that facilitate the generation of such hybrid clonal or allopolyploid vertebrates appears crucial for insights into the evolution of vertebrate sex, hybrid-origin gametogenetic aberrations, polyploidy and speciation. The advantages, disadvantages and consequences of sex chromosomes, including both undifferentiated and differentiated ones, present a promising area for future discoveries.

New sex chromosomes may have evolved more often within vertebrate lineages that already had established GSD in their ancestry than *de novo* from species with ESD. Such transitions from a pre-existing GSD to a derived GSD system are referred to as sex chromosome turnovers, whereby a new sex-determining locus emerges either on the same sex chromosomes or on an autosome, or by a translocation of an existing sex-determining locus (see below). Rapid rates of sex chromosome turnover have been documented in several lineages of anurans and teleosts [71–76], the groups where GSD is dominant and likely even the ancestral state [28,77]. Regardless of whether sex chromosomes evolved from an ESD/sequential hermaphroditic system without sex chromosomes, or in a GSD ancestor, an important question is:

## 3. Where do sex-determining loci come from?

In gonochoristic vertebrates, gonads start their development as bipotential primordial organs that later differentiate into ovaries or testes. It seems that sex determination has not only to activate one pathway, but at the same time to repress the alternative one [78]. In the classical scenario, only a single locus has to be changed to become the sex-determining locus and thus establish sex chromosomes. The traditional model starting from simultaneous hermaphroditism in angiosperms assumed the involvement of two linked loci [79]. Recently, the generality of this model was questioned for some plants, claiming that only one locus may have been involved [80,81]. In some cases, however, an existing single-locus system evolved likely through a two-loci stage [81].

In vertebrates, the sex-determining locus usually consists of a homologue of a gene from the gonadal differentiation networks that acquires a novel function as the switch to initiate ovarian or testicular differentiation [82]. A single known exception was detected in salmonids, where the sex-determining gene is a homologue of an immune-related gene; however, even in this case it strongly interacts with common players of the gonadal differentiation network [83,84]. Still, we advocate to continue the ‘hunt’ for sex-determining genes with promising techniques such as RNAseq of gonadal tissues in relevant embryonal stages and pool sequencing of panels of phenotypically contrasted sexed adults [16] to gain more robust knowledge on the identity of sex-determining genes and their origin. Functional approaches seem necessary too, but we have to keep in mind that a ‘proof of function’ by knockout-experiments to verify candidate sex-determining genes may be misleading since many genes of the gonadal differentiation networks may show the same effect as the ‘master’ sex-determining gene itself, i.e. resulting in experimental sex reversal [85]. This situation can be even more complicated if the knocked-out gene is also sex-linked and sex-specific.

A sex-determining locus mostly evolves as a new paralog (via duplication), or by allele differentiation. Both pathways are almost equally likely in vertebrates [86]. As alleles at a sex-determining locus can differ minimally—in an extreme case, only by a single nucleotide [87]—one can assume that the evolution of a novel sex-determining locus can be very easy and a minor genetic change appears sufficient for a major turnover in sex determination [11]. Traditionally, it was postulated that members of the sex differentiation pathways are very conserved across many vertebrate species or even non-vertebrate deuterostomes and protostomes [78,82], while sex-determining genes might be ephemeral and can be easily replaced by other sex-determining genes [88]. Nevertheless, the current state of knowledge suggests that even among closely related groups of organisms with indistinguishable gonadal development at the morphological, histological and cellular levels, molecular control of sex determination and gonad differentiation can differ substantially [78,82]. The detailed analysis of the relatively young sex-determining gene in medaka, *Oryzias latipes*, representing a radiation with evolutionarily unstable sex determination [72,73], revealed that the function of a new master sex-determining gene required several changes and substantial rewiring of interactions among members of the gonadal differentiation networks [55]. Adolfi *et al*. [89] argue that coevolution between a new sex-determining locus and other members of this network is required to restrict the function of the sex-determining locus to sex determination without negatively affecting other steps of sex differentiation and reproductive roles. Such changes can be an important pre-condition for the emergence of a new sex-determining trigger. The detection of the sex-determining locus may thus be only the tip of the iceberg of the underlying molecular changes, required for the transitions among sex determination systems. More detailed analyses of rewiring will help to understand how many changes are truly needed for a transition in sex determination and whether lineages with frequent transitions in sex chromosomes such as cichlids, sticklebacks, medaka and its relatives, ranid and pipid frogs [71–76] have specific preadaptations enabling such transitions in contrast to lineages with long stability of ESD such as sea turtles or crocodiles ([32,35]; case 8 in figure 2) or of sex chromosomes such as sturgeons [90] and several lineages of amniotes [35,91]. In lineages with stable sex chromosomes, their sex-determining locus might become an integral part of sexual development and be more resistant to replacements.

An intriguing way to gain insight into the changes required for the transitions in sex-determining loci is to test their function in related organisms with non-homologous sex determination. Roco *et al*. [92] did this by interspecies crossing experiments in the pipid frogs *Xenopus laevis* and *Xenopus tropicalis* and documented a pattern consistent with relatively simple and direct effects of sex-determining genes on gonadal and somatic development. Nevertheless, we do not know the interactions between the genes when the genetic background would be non-hybrid, which would require transgenic frogs. Would they show evidence for the need of coevolution/preadaptations to transitions in sex determination?

Further research into the role of sex-determining genes in primordial germ cells would be particularly interesting. In vertebrates and many other animals, the germline is established as a separate cell lineage early in development, and in many taxa primordial germ cells migrate to the developing gonads only later. Current evidence suggests that in some vertebrates, germ cells can influence whether the bipotential gonad will develop towards a testis or an ovary. For example in zebrafish, a complete absence or depletion of germ cells leads to the development of testes, regardless of the genotype of an embryo [93]. In medaka, it was demonstrated that XX and XY germ cells behave differently with respect to their mitotic activity and that the sex-determining gene on the Y is expressed there much earlier than in the somatic gonadal cells [94]. A specialized, typically maternally inherited chromosome restricted to germ cells (germline-restricted chromosome, GRC), eliminated during development from all somatic cells and from most spermatocytes, is likely present in all passerine birds [95]. There are no sexual differences among the zygotes in the number of GRCs, but male germline cells with two GRCs successively lose them, while female germ cells stably reproduce zygotic GRC karyotype. Potentially, GRC is preferentially segregated to eggs instead of polar bodies in female meiosis (meiotic drive), and it is a functional element of songbird germline genomes [96]. As far as is known, all birds have conserved ZZ/ZW sex chromosomes [97], and genes linked to the avian sex chromosomes contribute largely to cell-autonomous sexual differences in somatic tissues, including gonadal somatic cells in the medulla, as demonstrated in chicken, a non-passerine bird [98–100]. Chicken gonads (both ovaries and testes) can differentiate in the absence of germ cells [101]. Further research in passerines is needed to explore whether their sex development is controlled by multi-locus interactions between sex chromosomes and the specialized GRCs.

The degree of evolutionary plasticity of the gonadal differentiation pathway should be explored in future studies as it may explain why sex chromosomes are stable in some lineages, while prone to turnovers in others, which was traditionally attributed, e.g. to the rate of differentiation of sex chromosomes, which is the question we now address:

## 4. Which mechanisms are responsible for the reduction of recombination in sex chromosomes?

Sex chromosomes are sometimes easily detectable by cytogenetic methods, when they are heteromorphic (with Y and W chromosomes can be either expanded or miniaturized in size), heterochromatic or enriched (or in contrast depleted) in repetitive sequences [102,103]. In other cases, X and Y, and Z and W can be cytogenetically indistinguishable [87]. Traditionally, homomorphic sex chromosomes were understood to be poorly differentiated, while heteromorphic and highly differentiated. However, the terms homomorphy and heteromorphy specifically concern chromosome morphology (size and shape) as seen by conventional light microscopy, which is not necessarily correlated with the degree of differentiation at the sequence level. Heteromorphic sex chromosomes can be poorly differentiated and recombining across most of their length (e.g. neo-sex chromosomes), while homomorphic sex chromosomes can exhibit high sequence divergence [104]. The problem of the metrics of the degree of differentiation of sex chromosomes is also complicated by the difficulty in the distinction between neutral and functionally important differences. Although the search is ongoing for the best approach to measure the degree of sex chromosome differentiation and degeneration (as discussed in detail by Charlesworth [105]), it is clear that lineages can differ significantly in their rates of sex chromosome differentiation. Moreover, the evolution of sex chromosomes over time is rarely a unidirectional progression of accumulating divergence [106,107, this paper]. Nevertheless, the suppression of recombination is likely the most crucial process that initiates, progresses and thereby triggers structural changes, leading to differentiation of sex chromosomes.

For decades, the field was dominated by the classical adaptive hypothesis going back to R.A. Fisher [3] that sex chromosomes stop recombining due to sexually antagonistic selection. However, there is surprisingly little evidence for this hypothesis. Furthermore, despite the many unique characteristics of sex-linked regions, only minor attention has been given to neutral models for the cessation of recombination (but see [108]), an important requisite if we are to truly test adaptive hypotheses. Thus the question remains, are sex chromosomes indeed hotspots for the resolution of sexual conflict in the genome? Perrin [109] concludes that there is little support for a significant role of sexual antagonism in the evolutionary dynamics of sex chromosomes in ranid and hylid frogs. Comparative genomic studies demonstrate an extensive variability in the gene content of sex chromosomes across independently evolved sex determination systems, which are not particularly enriched with genes controlling sexual dimorphism. In fact, the genes that survived in the non-recombining regions of highly differentiated Y in mammals and sticklebacks and W chromosomes in birds and snakes are mainly dosage-sensitive, and their loss would be lethal [110–114]. On the other hand, Song *et al*. [74] demonstrate that the sex-biased expression of sex-linked genes occurred in parallel with the origin of independently evolved sex chromosomes in pipid frogs. Functional analyses seem necessary to uncover the contribution of this sex-biased expression to the resolution of sexual conflict, as it could also reflect mutations on the sex chromosome leading to loss of expression in one sex. In conclusion, it seems that sexual antagonism is either rare or hard to detect (present only under some environmental conditions, or detectable only under certain genetic backgrounds, i.e. subject to epistasis). Thus, sexual antagonism may not be a general and widespread driver of the cessation of recombination, and we should consider other models as well.

The probability of recombination is not equally distributed across genomes: there are hotspots and coldspots [115], and smaller chromosomes generally recombine more than larger ones [116]. The placement of a newly emerged sex-determining locus can affect the recombination rate around it. Moreover, heterozygosity and epigenetic changes can locally alter the recombination rate. And just a mutation leading to the emergence of a new sex-determining locus—and thus necessarily to heterozygosity [108]—can change the recombination rate in the linked region, particularly if this involves gene duplications and/or transposon activity. In addition, intrachromosomal rearrangements such as inversions, preventing recombination in a heterozygous state (always true if linked to the sex-specific allele of the sex-determining locus), seem much more frequent in some taxa than in others [117–119], which may contribute to different rates of sex chromosome differentiation in independently evolved systems.

Female and male meiosis also differ in several important aspects, very often in frequency and position of recombination, a phenomenon known as heterochiasmy. In vertebrates, females often recombine more evenly across the chromosome, but males more frequently near the chromosome tips [120]. Some regions recombine *a priori* less in a given sex and the position of the newly emerged male- or female-linked sex-determining gene might be crucial for the cessation of recombination. Phylogenetic reconstructions of recombination landscapes of ancestral autosomes and derived sex chromosomes, as well as detailed analyses of the mechanisms leading to recombination cessation (inversions, transposon activity, etc.), can be important for testing these hypotheses. Nevertheless, such analyses can be complicated by even rare incidence of sex reversals (driven by the environment or otherwise). The rate and position of recombination are often determined by the phenotypic rather than the genotypic sex. Therefore, sex chromosomes present in a sex-reversed individual may be subjected to the recombination pattern typical of the opposite phenotypic sex. According to the ‘fountain of youth’ hypothesis, this situation might produce newly recombined variants of sex chromosomes, potentially allowing purging of deleterious mutations from otherwise degenerating sex chromosomes and thus keeping sex chromosomes poorly differentiated [23–25].

## 5. Is the sex chromosome differentiation pathway truly unidirectional, i.e. leading from poorly to highly differentiated sex chromosomes, or even to the loss of Y and W?

The classical paradigm suggests that sex chromosomes will differentiate over time, leading to largely degraded Y and W chromosomes that eventually may even fully disappear. Although such a unidirectional pathway is described or depicted in nearly every paper on sex chromosome evolution (e.g. [8–15]), it is now becoming clear that it does not accommodate the enormous diversity of evolutionary pathways in the majority of lineages. Moreover, the unidirectional pathway may proceed at very different rates. In some lineages, sex chromosomes may remain at low levels of differentiation for long evolutionary periods (case 9 in figure 2), as is the case in the X and Y chromosomes of the pufferfish *Takifugu rubripes*, which differ at a single nucleotide, despite the sex determination gene being several millions of years old [87,121]. In birds, we find another example of sex chromosomes locked at a more advanced differentiation stage. Although ZZ/ZW sex chromosomes are homologous across birds [97] and thus of the same age, ostriches and most other paleognath birds have a much smaller non-recombining region, while neognath birds and palaeognath tinamas proceeded further in the process of sex chromosome differentiation [122–124].

Further contradicting the unidirectional scenario are evolutionary pathways of sex chromosomes that depart sideways into potentially interconnected trajectories or even evolve in a virtually reversed manner. Turnovers of sex chromosomes were described in many lineages of fish and amphibians with poorly differentiated sex chromosomes [71–76], resulting in two distinct pathways. The ancestral sex chromosomes can be replaced by a new sex determination system based on a new sex-determining locus on the same [125] or another chromosome pair [72–74], or the existing sex-determining gene can be translocated to another chromosome [126,127] (cases 14–16 in figure 2). Importantly, inferences about the homology of GSD systems should be based on knowledge on the sex-determining locus, not just on the identification of linkage groups representing sex chromosomes. A translocation of a sex-determining locus to other chromosome pair as documented for instance in salmonid fishes [83,84,126] even leads to a situation in which a homologous sex determination system is harboured by non-homologous sex chromosomes. And *vice versa*, that two taxa exhibit the same chromosome pair as sex chromosomes does not necessarily mean their sex determination systems are homologous: the same pair of autosomes can be independently co-opted for the function of sex chromosomes (e.g. [71,75,128]; for overview in amniotes see [34]), or there might be turnovers of sex determination systems by the emergence of a new sex-determining locus within the same sex chromosome pair [66,129] (cases 15, 18 and 20 in figure 2).

Even highly differentiated, cytogenetically detectable sex chromosomes can be replaced by poorly differentiated ones, as supported by results in basilisks and *Paroedura* geckos [130–132] (case 10 in figure 2). Ogata *et al*. [66] revealed that the heteromorphic sex chromosome systems probably returned to homomorphy through hybridization in the Japanese wrinkled frog *Glandirana rugosa*. In some cases, a simple system of two sex chromosomes changed into derived systems of three homologous sex chromosomes present in a population, e.g. in the African pygmy mouse *Mus minutoides*, where sex is determined by typical ancestral X, Y and derived X* causing feminization of X*Y individuals [133], or in the pipid frog *X. tropicalis*, in which sex is determined by the combination of Z, W and Y chromosomes [134], where the masculinizing Y chromosome evolved from the ancestral Z [129] (case 18 in figure 2). A more controversial question is whether sex chromosomes can be totally lost through the transition from GSD to ESD, where there are no consistent sexual differences in genotypes [12] (case 7 in figure 2). The only well-supported example comes from laboratory data on the bearded dragon *Pogona vitticeps*, in which the cytogenetically distinguishable ancestral W chromosome was lost in a single generation through the production of thermally induced, sex-reversed ZZ females, which mated with normal ZZ males and produced progeny with sex determination depending on the incubation temperature [135].

The classical paradigm predicts that, after the loss of recombination in a sex-linked region, the sequence will be lost, leading to heteromorphy. By contrast we now have numerous examples of genetic material being added onto the sex chromosomes [102]. For example, some sex-linked gene families have undergone massive copy number amplification of up to tens or hundreds of copies, likely driven by meiotic drive, as documented in mice and bovid sex chromosomes [136,137]. New material can also be added to sex chromosomes via translocations from autosomes. Such additions of previously autosomal material to both Z and W occurred in songbirds of the Sylvoidea superfamily [138,139] (case 13 in figure 2), and in a lineage of cichlid fish, the very large chromosome likely evolved through an addition of a B chromosome onto sex chromosomes [140]. A complex history was also reconstructed for human sex chromosomes, which contain the X-added region (added to the sex chromosomes in eutherian mammals but autosomal in marsupials), and the X-transposed region (transposed from the human X to the human Y after human–chimpanzee divergence) [141,142].

Another way for material to be added to the system of sex chromosomes is a fusion of the W or more often of the Y (as happened e.g. many times in placental mammals, iguanas and teleosts [143–145]) with an autosome, producing multiple neo-sex chromosomes (case 19 in figure 2), where the newly added parts behave as pseudoautosomal regions and can go through subsequent differentiation [133]. Notably, the size of Y and W can be expanded by the accumulation of repetitive sequences [102] (case 11 in figure 2), and repeats from these degenerated chromosomes can ‘contaminate’ and be amplified on X/Z as well [146,147] (case 12 in figure 2). Genes can also subsequently re-emerge in the degenerated parts of W and Y. In the latter case, a gene initially lost in the non-recombining region of Y and W can re-appear there owing to a recombination or translocation between sex chromosomes in otherwise non-recombining regions or by gene conversion [107,147,148].

The ultimate loss of degenerated Y or W owing to their total degradation is also controversial. The terminal stage of sex chromosome differentiation, when Y and W chromosomes disappear completely from the genome [8,9], leading to XX/X0 or ZZ/Z0 sex chromosome constitutions, can sporadically occur in some species. Specifically, few rodents have a derived system with XX/X0 sex chromosomes or the loss of Y chromosomes creating an X0/X0 situation in both sexes [9]; nevertheless, most of the genomic material was in these cases translocated to X chromosomes or autosomes [149]. In other cases, it is not clear whether the Y chromosome or a part of it fused with an autosome, creating a X_1_X_2_/X_1_X_2_Y system, though this could be tested by an analysis of male meiosis. XX/X0 and ZZ/Z0 systems were also reported in fish, but the current understanding of these systems is still limited [145]. However, the loss of Y/W chromosomes does not seem to be inevitable for all highly differentiated sex chromosomes. In lineages such as birds (age of sex chromosomes *ca* 110 Myr), trionychid turtles (120 Myr) and iguanas (120 Myr), the old and degraded Y/W chromosomes have persisted for tens of millions of years and have yet to be lost [150–152]. Analyses in primates demonstrated that there is a rapid loss of genes from Y chromosomes shortly after the formation of each evolutionary stratum, but that gene loss slows over time until it reaches a level at which conservation is maintained, likely through purifying selection [153]. Bellott *et al*. [110] modelled that the loss of Y chromosomes is far from being inevitable more generally in mammals. Thus, losses of degenerated Y and W seem rare, they are difficult to predict and it seems that they represent a less likely pathway of sex chromosome evolution. Indeed, what many call ‘degenerated’ sex chromosomes, might instead be seen as specialized as they almost always contain genes important or essential for the heterogametic sex or dosage-sensitive genes requiring two functional copies in diploid genomes. Such genes make it less likely that old sex chromosomes can or will be lost. Moreover, even degenerated, gene-depleted Y and W chromosomes can be conserved to ensure proper chromosome pairing and segregation during meiosis. In some invertebrate lineages, such as caddisflies, moths and butterflies or spiders, ZZ/Z0 and XX/X0 sex chromosomes might be ancestral, while W and Y sex chromosomes evolved later in certain sublineages [154,155]. If ZZ/Z0 and XX/X0 sex chromosome systems evolved via aneuploidy, i.e. instantaneous loss of one copy of ancestral autosome from the genome, this situation might not be terminal, but might instead be just the initial step in sex chromosome evolution.

## 6. Is there variation in sex chromosome differentiation between male (XX/XY) and female (ZZ/ZW) heterogamety?

The classical paradigm is principally identical for sex chromosome differentiation under male and female heterogamety and assumes that the processes are comparable. But is that really the case, or do we need different scenarios for each type of heterogamety? Theory predicts that a Y chromosome should differentiate faster than a W chromosome, i.e. the classical sex chromosome differentiation pathway (figure 1) should be shorter in time under male than under female heterogamety. It could be caused by higher mutation bias and stronger selection in males, or a smaller effective population size of the Y chromosome with decreasing ratio of reproducing males to females in a population [156–158]. However, phylogenetic comparisons do not strongly support these predictions. Namely, thanks to the application of genomic techniques such as RADseq in recent years, several counterexamples from poorly differentiated sex chromosomes have emerged. For instance, in squamate reptiles, it was found that both snake lineages with female heterogamety possess heteromorphic sex chromosomes, while the two snake lineages with male heterogamety have homomorphic, cytogenetically indistinguishable sex chromosomes [159,160], although there is no evidence that the female heterogametic systems should be considerably older. A similar pattern can be found in lacertoidean lizards, where tegus, whiptails and spectacles lizards (families Teiidae and Gymnophthalmidae), presumably with male heterogamety, have only poorly differentiated sex chromosomes, while closely related true lizards (Lacertidae) from their sister clade exhibit female heterogamety with highly differentiated sex chromosomes. The same trend was observed within chameleons (reviewed in [91,160]). Likewise, sex chromosomes seem to differentiate at a faster rate under female than male heterogamety in teleost fishes [145]. Ancient, but still quite poorly differentiated XX/XY sex chromosomes were also demonstrated in skinks [91,161] and ZZ/ZW in sturgeons [90]. Thus, counter to theoretical predictions, sex chromosomes do not seem to differentiate faster and/or are subjected to more turnovers under male than female heterogamety. Therefore, we should re-examine why differentiation may proceed faster in some lineages regardless of the type of heterogamety.

A dichotomy in the gene dose regulatory mechanisms between XX/XY and ZZ/ZW systems was also postulated. The differentiation of sex chromosomes leads to unequal numbers of functional copies of many genes between the sexes. This imbalance has to be dealt with at the cellular level as the protein production in a cell is generally affected by the number of transcribed gene copies, and cell physiology and differentiation require proper stoichiometric ratios of interacting proteins [162,163]. Some lineages have evolved dosage compensation, the epigenetic mechanism that restores the expression of the X- or Z-specific genes in the heterogametic sex to the expression levels prior to sex chromosome differentiation [2,164]. Other lineages equalize the expression levels of the X- or Z-specific genes between the sexes, but not to the ancestral levels, i.e. possess incomplete compensation with dosage balance. The third documented type of a gene dose regulatory mechanism is an incomplete compensation without balance, also referred to as incomplete or partial dosage compensation, where expression of sex-specific loci remains lower in the heterogametic sex relative to the homogametic sex [164]. Nevertheless, we should also keep in mind that the gene dose regulatory mechanisms can be tissue- and age-specific [165]. A complete (global) or nearly complete dosage compensation or at least dosage balance between sexes was often found in lineages with XX/XY sex determination. On the other hand, an incomplete compensation without balance seems to be common in lineages with female heterogamety [164,166–168]; however, the reasons for these differences were not clear. The suggested explanations include mostly adaptive processes such as a stronger selection for dosage balance in lineages with male heterogamety owing to faster degeneration of the Y than the W (as a result of higher mutation rates in males) or differences in effective population size between sexes (owing to differences in the strength of sexual selection and different strength of sexually antagonistic selection in males and females). However, notable exceptions to the rule were recently found, and an overview of gene dose regulatory mechanisms does not support any clear differences between animal lineages with male versus female heterogamety [169].

Recent studies demonstrated that XX/XY and ZZ/ZW sex chromosomes in vertebrates notably differ in the frequency of neo-sex chromosome systems by fission of sex chromosomes or predominantly fusion with an autosome [143–145]. For example, multiple neo-sex chromosomes evolved independently more than 20 times in mammals and around 15 times in iguanas with male heterogamety, whereas only six times in caenophidian snakes and maybe just once in birds with female heterogamety [143,170]. An explanation of this pattern is unclear (for recent overview see [145]), but it was suggested that it can reflect a faster differentiation rate of the Y in comparison to the W owing to higher mutation bias or stronger selection in males (e.g. [144]). Alternatively, it was proposed that the differing tendencies for the formation of multiple neo-sex chromosomes between male and female heterogamety might be explained by differential involvement of sex chromosomes in female meiosis, particularly in female meiotic drive, i.e. the bias in the segregation of chromosomes into the egg nucleus versus polar bodies. According to this hypothesis, the structural changes like fissions/fusions are subjected to meiotic drive during female meiosis on chromosomes X, Z and W, but not on the Y chromosome, which occurs only in males [143,171]. The female meiotic drive may operate on centromeres [172,173] and fusions and fissions of chromosomes can alter just the centromere structure. Interestingly, multiple neo-sex chromosomes of the types Z_1_Z_1_Z_2_Z_2_/Z_1_Z_2_W, ZZ/ZW_1_W_2_ and other types and even ZZ/Z0 sex chromosomes are common in butterflies and moths (Lepidoptera) and their sister group, the caddisflies (Trichoptera) [174]. It is possible that this pattern corresponds to their holocentric chromosomes, which might obey the female meiotic drive of centromeres [175]. The hypothesis on the driving centromeres in organisms with monocentric chromosomes and asymmetric female meiosis predicts that female meiotic drive will more strongly select for homogenization of the centromere structure of sex chromosomes under female heterogamety. Unfortunately, to our knowledge, there is no systematic comparison of centromeres of sex chromosomes between female and male heterogamety, which is a clear avenue for further research.

## 7. Were particular genomic regions more frequently co-opted for the role of sex chromosomes?

In their insightful paper, Marshall Graves & Peichel [128] debated whether the evolution of sex chromosomes is random, or whether certain syntenic blocks have a higher chance to become a part of sex chromosomes. The non-random co-option of certain genomic regions could reflect a limited pool of genes involved in gonadal development (‘usual suspects’ such as *amh*, *ar*, *dmrt1* or *sox3*) that can evolve as a master sex-determining gene by turning their syntenic blocks into sex chromosomes. Indeed, certain syntenic blocks seem to emerge more often as sex chromosomes in ranid frogs [71] and amniotes, but at least in amniotes, this non-random pattern is not particularly strong [34]. The numerous exceptions can be caused by the emergence of a sex-determining locus via duplication within a different syntenic block from the original one with the ‘usual suspect’ gene, or by the existence of more genes with a potential to become sex-determining genes than we assume.

In addition, selection can favour the location of a sex-determining gene in a region enriched by genes with sexually antagonistic effects [176]. However, as argued by Perrin [109], there is little role for sexual antagonism in the evolution of sex chromosomes in ranids and it is thus unlikely that the non-random co-option observed in this group would reflect the sexually antagonistic selection. Likewise, Lichilín *et al*. [75] did not find evidence that sex-linked genes contribute exceptionally to sexual dimorphism in a clade of cichlid fish. In this group, chromosomes that became sex-linked were not enriched in genes with sex-biased expression before their recruitment as sex chromosomes [75], which might imply only a minor or no role of sexual antagonism for the co-option of chromosomes as sex chromosomes. It could also mean that there is no further benefit for a chromosome on which conflict was resolved by other mechanisms (e.g. sex-specific expression) to transition to a sex chromosome. On the other hand, the hypothesis that sexual antagonism can favour the location of a sex-determining gene in a region enriched by genes with sexually antagonistic effects received support by non-random fusions of certain autosomes with sex chromosomes in iguanas [177] and songbirds [139]. Particularly in songbirds, one region fused to the ancestral ZZ/ZW was found to be enriched in genes with predicted sex-related functions [139]. Non-random fusions can, however, be explained by the close physical proximity of particular chromosomes in the nucleus, as recently supported by the analyses of the multiple neo-sex chromosomes formation in platypus [178]. In fact, new data in iguanas [177] confirm more frequent involvement of certain chromosomes in sex chromosome formation, but at the same time do not reveal a connection between the sex chromosome–autosome fusions and the evolution of recombination rate, which would be important for a role of sex chromosomes in the resolution of intralocus sexual conflict. The reasons for the non-random fusion of certain genomic parts to sex chromosomes should be explored in the future, using comparative data.

## 8. Conclusion

Exactly 130 years after Henking's observation of the enigmatic ‘X element’ [179], over 115 years after McClung and Stevens hypothesized that these ‘accessory chromosomes' determine sex [180,181] and more than 100 years after the early version of the classical paradigm on sex chromosome evolution [1], this review alongside the two adjoined theme issues of the *Phil. Trans. R. Soc. B.* celebrates the contribution of vertebrate research to the great progress made in our understanding of sex chromosome evolution. However, despite this substantial progress, we still lack an understanding of why sex chromosomes emerge and differentiate at highly unequal rates in different lineages, and why they are even absent in several animal lineages. Thus we highlight the need for additional data on the germline and the whole gonadal differentiation networks to further explore the evolution of sex-determining genes. Likewise, research should focus on structural changes accompanied by sex chromosome formation, including the mechanisms responsible for the suppression of recombination. Most likely, we will also have to revise why the empirical results on sex chromosome differentiation rate and the evolution of gene dose regulatory mechanisms between male versus female heterogamety do not extensively follow theoretical expectations. The consequences of differentiated/degenerated sex chromosomes for individual fitness, lineage evolvability and diversification will also be important research topics.

Although the classical paradigm is ingenious and insightful, driving decades of research, its unidirectional scenario, aimed primarily to explain the evolution of highly differentiated sex chromosomes, has turned out to be too simplistic. We admire this model for its intellectual beauty, historical importance and power to stimulate research, but we consider it now as an overidealized model requiring expansion. Sex chromosome evolution is truly complex, and far from unidirectional. Instead, it is a multi-faceted process with many side roads, stability of particular ‘stages’ in certain lineages, alterations and even apparent reversals, virtually breaking Dollo's law of irreversibility. We thus conclude that the realistic picture is not simply linear. However, we also doubt that sex chromosome differentiation is linear with simple loops as depicted by Abbot *et al*. [106] or cyclic as depicted, e.g. in Furman *et al*. [107]. Instead, we think that the process principally represents a network of evolutionary trajectories branched off to potentially endless outcomes with a possibility to freeze for a long time and even reverse to certain states locally (figure 2).

## Endnote

1 For example, Muller [1, p. 483]: ‘Moreover, the ‘degenerative’ changes in these cases could be much more extensive than in cases of balanced lethals, because the Y and W chromosomes were more completely protected by their homologues from the action of selection. This is because their homologues (X and Z, respectively) themselves remained quite normal, owing to the fact that they were subjected to selection when in the homozygous sex (XX or ZZ). Recessive changes could consequently be established in any locus of Y and W, whereas in cases of balanced lethals, where both homologues might become involved, only half of either chromosome might, on the average, degenerate. (…)’; p. 485: ‘after an extensive degeneration has taken place in either member of a pair of balanced chromosomes, many recessive changes in the other member will be able to ‘show’, just as recessive mutants in the X chromosome can now manifest themselves in the XY male, owing to the ‘degenerate’ condition of the Y’.
